# Growth Hormone and Disease Severity in Early Stage of Multiple Sclerosis

**DOI:** 10.1155/2013/836486

**Published:** 2013-10-23

**Authors:** M. Gironi, C. Solaro, C. Meazza, M. Vaghi, L. Montagna, M. Rovaris, A. P. Batocchi, R. Nemni, R. Albertini, M. Zaffaroni, M. Bozzola

**Affiliations:** ^1^INSPE, Ospedale San Raffaele, Milano, Italy; ^2^CAM, Centro Polidiagnostico, Monza, Italy; ^3^Dipartimento di Neurologia, Ospedale Micone, Genova, Italy; ^4^Fondazione IRCCS San Matteo, Dipartimento di Medicina Interna e Terapia Medica, Università di Pavia, Pavia, Italy; ^5^Dipartimento di Scienze Clinico-Chirurgiche, Diagnostiche e Pediatriche, Università di Pavia, Pavia, Italy; ^6^Fondazione IRCCS, S. Maria Nascente, Don Gnocchi, Milano, Italy; ^7^Dipartimento di Neuroscienze, Università Cattolica, Roma, Italy; ^8^Laboratorio di Biochimica Clinica, Fondazione IRCCS San Matteo, Pavia, Italy; ^9^Centro Studi Sclerosi Multipla, Ospedale S. Antonio Abate, Gallarate, Italy

## Abstract

Evidence suggests that neurohormones such as GH and IGF-I are involved in the neuroreparative processes in multiple sclerosis (MS). GH and IGF-I blood levels in naïve MS patients with different disease courses were investigated in this study. Serum GH and IGF-I in untreated MS patients (*n* = 64), healthy controls (HC, *n* = 62), and patients affected by other neurological diseases (OND, *n* = 46) were evaluated with a solid-phase-enzyme-labeled-chemiluminescent-immunometric assay. No differences were detected in GH across MS, OND, and HC (MS = 0.87 ± 1.32 ng/mL; OND = 1.66 ± 3.7; and HC = 1.69 ± 3.35; *P* = 0.858) when considering gender, disease duration, and disease course. However, GH was lower (*P* = 0.007) in patients with more severe disease (expanded disability scale score, EDSS ≥ 4.0) compared with milder forms (EDSS < 4). IGF-I l did not differ across the 3 groups (*P* = 0.160), as far as concern disease course, disability, and gender were. Lower IGF-I levels were detected in subjects older than 50 years compared to younger ones for all 3 groups. Lower GH was detected in patients with more severe MS, and age was confirmed as the main factor driving IGF-I levels in all subjects. These findings, relying on the natural course of the disease, could help in shedding lights on the mechanisms involved in autoreparative failure associated with poorer prognosis in MS.

## 1. Introduction

Multiple sclerosis (MS) is a chronic demyelinating disease of the central nervous system with an unpredictable time course. Among the plethora of factors affecting the clinical heterogeneity of MS, autoreparative mechanisms are of particular importance. Remyelination is largely known to occur in MS [1], but it is still unclear why its adequacy differs so largely among patients. Many factors have been proposed to influence remyelination, including several neuroendocrine factors [2, 3]. Unresponsiveness to these factors and/or their insufficient release could possibly be involved in reparative mechanism failure, and studies focusing on these molecules have attracted a great deal of attention.

Growth hormone and IGF-I have been recognised as factors that can affect survival of myelin and central nervous system (CNS) cells [3, 4]. Several studies [2–4] have focused on these growth factors, unfortunately with equivocal results.

Heterogeneity is not largely dependent on the different methodologies used but also on the disease's natural history. Growth factor bioavailability can vary in the different phases of the disease leading to a permissive or, on the contrary, an inadequate microenvironment supporting remyelination. Moreover, another putative confounding factor could be the different treatments known to influence neurohormone secretion (e.g., glucocorticoids).

The aim of the present study was to investigate GH and IGF-I blood levels in a population of treatment naïve MS patients at different phases of the disease. Two different control groups were used, namely, healthy control (HC) subjects and other neurological disease (OND) patients.

## 2. Materials and Methods

### 2.1. Subjects

Sixty-four therapy-naïve MS patients, 46 age-matched subjects affected by OND, and 62 healthy controls HC were recruited as outpatients from June 2009 to June 2011 at S. Maria Nascente, Fondazione Don C. Gnocchi (Milan Italy), at Ospedale S. Antonio Abate (Gallarate, VA Italy), at Ospedale Micone (Genova Italy), and at the Department of Neuroscience of Cattolica University (Rome Italy). The study protocol was approved by the local Ethics Committees of the respective institutions in accordance with the Declaration of Helsinki (1964). Eligible subjects signed a written informed consensus. MS patients satisfied the Polman criteria [5] and were in a clinically stable phase for at least two months earlier and not treated with immunomodulatory or immunosuppressive drugs. The OND group included patients affected by chronic neurological diseases different from demyelinating disorders (posttraumatic haemorrhage, stroke, mild cognitive impairment, and headache). Exclusion criteria included treatment with steroids, *β*-blockers, selective serotonin reuptake inhibitors (SSRI), and benzodiazepines (known to influence GH and IGF-I levels).

Disease course was classified as relapsing-remitting (RR: *n* = 22), secondary progressive (SP: *n* = 23), or primary progressive (PP: *n* = 19). All MS patients were evaluated with the expanded disability severity scale (EDSS). Subjects were matched for age and sex (*P* = 0.786; *P* = 0.640, Table 1).

Blood samples were collected in the morning (8 am–10 am) in a fasting state and delivered to the central laboratory of the IRCCS San Matteo Foundation, within 12 hours. Serum samples were obtained by centrifugation and stored at −20°C until assays could be performed.

### 2.2. Immunometric Assays

Serum GH and IGF-I were assayed with a fully automated immunochemistry analyser, immulite 2000 (Siemens Diagnostics). Methods for assaying GH and IGF-I were based on a solid phase, two-site immunometric sandwich assay with a chemiluminescent signal. The method for GH assay is characterized by an analytical sensitivity of 0.01 ng/mL and a linearity range (reportable range) from 0.05 to 40 ng/mL. The intra- and interassay coefficients of variation for GH were 5.3%–6.5% and 5.7%–6.1% for a quality control range of 1.7–31 ng/mL and 3.0–18 ng/mL, respectively. The intra- and inter-assay coefficients of variation for IGF-I were 3.9%–2.4% and 5.1%–4.8% for a quality control range of 77–1,358 ng/mL, respectively.

### 2.3. Statistical Analysis

All statistical analyses were performed using the statistical package for the Social Sciences (SPSS), version 15.0, for Windows (SPSS, Chicago, IL, USA).The normality of the distribution for all variables was assessed by the Kolmogorov-Smirnov test. The Student's *t*-test and ANOVA were used for normally distributed variables. Neither serum GH nor IGF-I blood levels were normally distributed as evidenced with the Kolmogorov-Smirnov test (serum GH: *P* = 0.000; serum IGF-I: *P* = 0.043). Accordingly, for both GH and IGF-I, differences between two groups were assessed with the Mann-Whitney *U* test. Differences between more than two groups were assessed with the Kruskal-Wallis test. A *P* value <0.05 was considered statistically significant.

## 3. Results

The characteristics of MS patients, OND, and the HC group are summarized in Table 1.

IGF-I did not significantly differ in MS compared with HC and OND subjects (*P* = 0.160, Table 2). No group differences were associated with different disease courses neither for GH (PP = 0.96 ± 1.71 ng/mL; SP = 0.88 ± 1.26 ng/mL; and RR = 0.79 ± 1.05 ng/mL; and *P* = 0.662) nor for IGF-I (PP = 161.86 ± 62.66 ng/mL; SP = 135.23 ± 47.82 ng/mL; and RR = 163.44 ± 53.98 ng/mL; *P* = 0.182).

Moreover, as far as gender was concerned, significantly higher levels of GH were detected in females compared with males for MS, OND, and HC groups (Table 2). A trend in lower levels of GH in female MS patients compared with female OND and HC subjects was also observed (Table 2).

For all studied groups (MS, OND, and HC), IGF-I was lower in older subjects (≥50 years) compared with younger subjects (<50 years) (Table 2). No such difference was detected for GH levels (Table 2).

Importantly, we also investigated a possible relationship between GH and IGF-I levels and severity of disease. We considered 500 meter walking autonomy (scored EDSS = 4), a clinically significant progression marker. Accordingly, MS patients were stratified into different groups. Group A: EDSS ≥ 4 within the first 10 years of disease; group B: EDSS < 4 within the first 10 years of disease; group C: EDSS ≥ 4 with disease duration longer than 10 years; and group D: EDSS < 4 with disease duration longer than 10 years.

**Figure 1:**
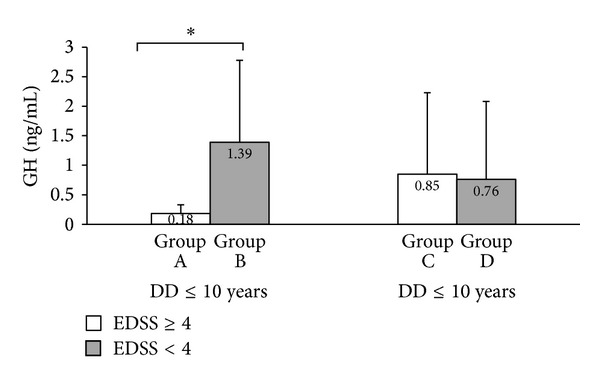
Serum GH levels in MS patients according to disease duration and EDSS score. Values are expressed as mean ± SD; *P < 0.05 (nonparametric test Mann-Whitney *U*).

Lower GH levels were detected in patients with EDSS ≥ 4 within the first 10 years of the disease compared to patients with EDSS < 4 within the first 10 years of the disease (group A: 0.18 ± 0.15 ng/mL; group B: 1.39 ± 1.39 ng/mL; *P* = 0.007, Figure 1). The number of females in group B was not significantly different from the number of females in group A (*χ* ^2^ = 2.48; *P* = 0.115).

Interestingly, there were no differences for longer disease duration (more than 10 years) (group C: 0.85 ± 1.38; group D: 0.76 ± 1.32 ng/mL; *P* = 0.558, Figure 1).

Considering IGF-I, no differences were found when comparing patients stratified according to the same criteria of disease duration and MS severity (data not shown).

## 4. Discussion

The phenomenon of remyelination is regulated by several mechanisms and influenced by the bioavailability of many molecules. Much attention has been focused on growth factors, such as GH and IGF-I, which are well known to positively affect remyelination. IGF-I is a potent neuroprotective factor for neurons and oligodendrocytes [3, 6] involved in oligodendrocyte precursor cell (OPC) proliferation and differentiation [1] and in myelin synthesis [3, 6]. Its secretion is induced by GH which is a neuroprotective molecule itself for adult myelin maintenance and endowed with antiapoptotic effects [4]. Shedding light on their roles *in vivo* could prove to be noteworthy, since these growth factors are now available as substitutive therapy [7] to be tailored to the peculiar need of an individual disease.

Our study did not detect any significant differences concerning GH and IGF-I levels in MS patients. Interestingly, however, we found lower GH levels in MS patients with a higher disability score (EDSS ≥ 4.0) during the first 10 years of disease than in patients with a milder disease course (no walking limitations). Importantly, this difference is present within the first 10 years of the disease, but it disappears after this time window. We believe these findings are interesting for several reasons. First, remyelination has been shown to occur over the entire disease course, but the phenomenon is most evident during the first years of disease [1]. It is reasonable to postulate that when myelin and oligodendroglial turnover increase, as it happens in the early phase of MS, GH may positively modulate repair mechanisms. It is also possible to speculate that carrying a higher level of GH during this early phase might therefore confer protection to OPC survival, establishing a more favourable disease course. Secondly, the lack of difference in serum IGF-I, according to the same stratification criteria, strengthens the role of GH as a neuroprotective factor itself [4], not just a mediator of IGF-I release. Thirdly, MS patients were all untreated; thus, confounding factors due to immune-modulatory (suppressive) treatment can reasonably be excluded. Accordingly, the results of the present study cannot be linked to different dosages or types of therapy in more disabled patients but crucially rely on natural mechanisms of the disease.

Another noteworthy finding is the detection of lower levels of IGF-I in patients older than 50 years. We confirmed the well-known [8] inverse correlation between age and IGF-I. Importantly, such a correlation was observed even in MS patients. It is conceivable that lower levels of IGF-I may hinder reparative processes. According to this hypothesis, the resulting inefficiency in reparative processes could enhance disease progression in older patients and would explain the worse prognosis reported in the literature associated with late disease onset [9].

Although not statistically significant, we have shown lower GH levels in MS females compared with control and OND females. Females are well known to be GH resistant and have higher GH serum levels than males [10]. This gender difference was observed also in MS patients, but between males and females MS, the difference was weaker than that shown for OND or HC. Female-GH resistance, when not overcome by higher GH level (as in healthy and OND females), might be speculated as one of the factors involved in female-MS prevalence [11].

The main drawback of our study was the small number of patients enrolled in each disease class. This would also explain the lack of statistical significance reported. In addition, several other factors can explain our results, indirectly modulating the GH/IGF-I axis. For example, the role of IGF binding proteins (IGFBPs) has been investigated, as these proteins are primarily responsible for transport of IGF-I [3] to target tissues, and they play an inhibitory role in IGF-I-driven myelination.

Recently, Lanzillo and colleagues [2] did not find a difference in IGF-I per se between HC and MS but detected a lower IGF-I/IGFBP-3 ratio in MS compared with controls. Interestingly, higher levels of IGFBP-3 in more disabled patients (with higher EDSS at 10 years of disease) were found, suggesting the reduced bioavailability of IGF-I (more than a lower IGF-I level) as a possible pathogenetic factor. Although our study was not designed to investigate IGF-I bioavailability, hence; we did not measure IGFBP, our results could be in agreement with those of the authors. The lack of different values for IGF-I in higher EDSS patients, as we disclosed for GH, could be associated with higher IGFBP (lower bioavailability). Unlike GH, IGF-I bioavailability more than IGF-I level might be relevant to MS susceptibility.

## 5. Conclusions

In conclusion, we reported a lower level of GH for higher cumulative disability and lower IGF-I for older patients. Notwithstanding several biases of the study (no cerebrospinal fluid analyses available, relatively small groups), our findings are noteworthy because they correlate with the natural course of the disease (not biased by treatment confounding factors). They sound to suggest that blood GH and IGF-I levels should not be considered as biomarkers of disease, but rather prognostic biomarkers. These findings should encourage larger studies to dissect further the involvement of GH and IGF-I in MS mechanisms.

## Figures and Tables

**Table 1:** Demographic and clinical characteristics of the MS, OND patients, OND and HC.

	MS (*n* = 64)	OND (*n* = 46)	HC (n = 62)	*P*
Age (mean ± SD)	50.21 ± 8.08	48.48 ± 15.41	48.87 ± 7.14	0.640
Male : female	27 : 37	20 : 26	23 : 39	0.786
EDSS (mean ± SD)	4.57 ± 2.17	—	—	—
PP (*n*)	19	—	—	—
SP (*n*)	23	—	—	—
RR (*n*)	22	—	—	—

RR: relapsing-remitting; SP: secondary progressive; PP: primary progressive, EDSS: expanded disability scale score.

**Table 2:** GH and IGF-I levels in MS patients, OND and HC subjects, according to sex and age.

GH (ng/mL)								IGF-I (ng/mL)						
	Tot	Sex			Age			Tot	Sex			Age		
		M	F	*P* ^§^	<50 years	≥50 years	*P* ^§^		M	F	*P* ^§^	<50 years	≥50 years	*P* ^§^
MS	0.87 ± 1.32 (*n* = 64)	0.50 ± 0.96 (*n* = 26)	1.15 ± 1.48 (*n* = 37)	**0.005**	0.85 ± 1.27 (*n* = 31)	0.89 ± 1.38 (*n* = 33)	0.898	152.8 ± 55.4 (*n* = 64)	145.3 ± 49.0 (*n* = 26)	158.0 ± 60.3 (*n* = 37)	0.434	171.7 ± 61.2 (*n* = 31)	135.2 ± 43.2 (*n* = 33)	**0.005**
OND	1.66 ± 3.7 (*n* = 46)	0.49 ± 0.74 (*n* = 20)	2.55 ± 4.72 (*n* = 26)	**0.002**	1.39 ± 2.53 (*n* = 24)	1.95 ± 4.71 (*n* = 22)	0.509	162.3 ± 77.9 (*n* = 46)	161.8 ± 74.5 (*n* = 20)	162.7 ± 81.8 (*n* = 26)	0.706	190.3 ± 73.3 (*n* = 24)	131.8 ± 72.4 (*n* = 22)	**0.004**
HC	1.69 ± 3.35 (*n* = 62)	0.45 ± 0.88 (*n* = 23)	2.42 ± 4.00 (*n* = 39)	**0.005**	2.24 ± 4.17 (*n* = 33)	1.06 ± 1.94 (*n* = 29)	0.374	136.4 ± 47.4 (*n* = 62)	141.1 ± 45.7 (*n* = 23)	133.7 ± 8.69 (*n* = 39)	0.512	147.5 ± 43.5 (*n* = 33)	123.8 ± 49.1 (*n* = 29)	**0.012**
*P* ^§§^	0.858	0.910	**0.545**		0.783	0.840		0.160	0.606	0.140		0.061	0.362	

*P* ^§^ Mann-Whitney *U*-test was used to compare MS versus OND and MS versus HC; *P* ^§§^ Kruskal-Wallis test was employed. GH in MS female shows a trend for lower value than in OND and HC females. Values are expressed as means ± SD. MS: Multiple Sclerosis; OND: other neurological disease; HC: healthy controls; M: male; F: female.

## References

[1] Franklin RJM (2002). Why does remyelination fail in multiple sclerosis?. *Nature Reviews Neuroscience*.

[2] Lanzillo R, Di Somma C, Quarantelli M (2011). Insulin-like growth factor (IGF)-I and IGF-binding protein-3 serum levels in relapsing-remitting and secondary progressive multiple sclerosis patients. *European Journal of Neurology*.

[3] Wilczak N, Ramsaransing GSM, Mostert J, Chesik D, De Keyser J (2005). Serum levels of insulin-like growth factor-I and insulin like growth factor binding protein-3 in relapsing and primary progressive multiple sclerosis. *Multiple Sclerosis*.

[4] Poljakovic Z, Zurak N, Brinar V, Korsic M, Basic S, Hajnsek S (2006). Growth hormone and insulin growth factor-I levels in plasma and cerebrospinal fluid of patients with multiple sclerosis. *Clinical Neurology and Neurosurgery*.

[5] Polman CH, Reingold SC, Edan G (2005). Diagnostic criteria for multiple sclerosis: 2005 revisions to the ‘McDonald Criteria’. *Annals of Neurology*.

[6] Hua K, Forbes ME, Lichtenwalner RJ, Sonntag WE, Riddle DR (2009). Adult-onset deficiency in growth hormone and insulin-like growth factor-I alters oligodendrocyte turnover in the corpus callosum. *Glia*.

[7] Frank JA, Richert N, Lewis B (2002). A pilot study of recombinant insulin-like growth factor-1 in seven multiple sclerosis patients. *Multiple Sclerosis*.

[8] Elmlinger MW, Kühnel W, Weber MM, Ranke MB (2004). Reference ranges for two automated chemiluminiscent assays for serum insulin-like growth factor I (IGF-1) and IGF-binding protein 3 (IGFBP-3). *Clinical Chemistry and Laboratory Medicine*.

[9] Trojano M, Pellegrini F, Fuiani A (2007). New natural history of interferon-*β*-treated relapsing multiple sclerosis. *Annals of Neurology*.

[10] Van den Berg G, Veldhuis JD, Frölich M, Roelfsema F (1996). An amplitude-specific divergence in the pulsatile mode of growth hormone (GH) secretion underlies the gender difference in mean GH concentrations in men and premenopausal women. *Journal of Clinical Endocrinology and Metabolism*.

[11] Voskuhl RR, Gold SM (2012). Sex-related factors in multiple sclerosis susceptibility and progression. *Nature Reviews Neurology*.

